# Antimicrobial Activity of Silver Nanoparticles Synthesized Using Ocimum tenuiflorum and Ocimum gratissimum Herbal Formulations

**DOI:** 10.7759/cureus.54994

**Published:** 2024-02-26

**Authors:** Remmiya Mary Varghese, Aravind Kumar S, Rajeshkumar Shanmugam

**Affiliations:** 1 Department of Orthodontics and Dentofacial Orthopedics, Saveetha Dental College and Hospitals, Saveetha Institute of Medical and Technical Sciences, Saveetha University, Chennai, IND; 2 Department of Pharmacology, Saveetha Dental College and Hospitals, Saveetha Institute of Medical and Technical Sciences, Saveetha University, Chennai, IND

**Keywords:** herb, antimicrobial herbs, time kill assay, silvernanoparticle, nanotechnology

## Abstract

Background

The exploration of green synthesis for silver nanoparticles using diverse plant sources has gained significant attention. This study specifically investigates the use of *Ocimum tenuiflorum* and *Ocimum gratissimum*, known for their antibacterial properties, in synthesizing silver nanoparticles. The primary aim of this study is to evaluate the antimicrobial efficacy of silver nanoparticles synthesized using a herbal formulation composed of *Ocimum tenuiflorum* and *Ocimum gratissimum* against different oral pathogens.

Materials and methods

The process involved the combination of herbal extracts from *Ocimum tenuiflorum* and *Ocimum gratissimum *with a silver nitrate solution leading to the synthesis of silver nanoparticles. The formation of silver nanoparticles was confirmed by ultraviolet and visible absorption spectroscopy. The obtained silver nanoparticles were used to study their antimicrobial activity. Antimicrobial activity was assessed using the agar well diffusion method against pathogens including *Streptococcus *mutans, Enterococcus faecalis, C. albicans, Lactobacillus acidophilus, and S. aureus**. The zone of inhibition quantified antimicrobial effectiveness. A time-kill curve assay evaluated bactericidal properties and the concentration-dependent relationship between silver nanoparticles and the net growth rate of oral pathogens.

Results

Statistical analysis was done to compare measures such as mean, standard deviation, and percentages. The antimicrobial assessment demonstrated that 100 μg/mL of silver nanoparticles exhibited the highest efficacy against *S. mutans, S.* *aureus, E. faecalis*, and *Lactobacillus *sp*.* For *C. albicans*, all concentrations of silver nanoparticles and the control plant extract displayed similar antimicrobial activity. The time-kill assay illustrated effective inhibition at 100 μg/mL against all tested pathogens, including *S. mutans, S. aureus, E. faecalis, C. albicans, and Lactobacillus *sp*. *The result showed positive inhibitory activity of silver nanoparticles against all tested bacterial strains.

Conclusion

The significant antimicrobial efficacy of green-synthesized silver nanoparticles positions them as promising candidates for dental applications. Their demonstrated bactericidal and fungicidal activities suggest potential use as effective dental antimicrobial agents, opening avenues for innovative solutions in oral healthcare.

## Introduction

In the context of viral infections, including the formidable challenges posed by the COVID-19 pandemic, nanotechnology has emerged as a beacon of hope. The development of antiviral and antimicrobial formulations showcases the adaptability of nanotechnological solutions in addressing contemporary healthcare challenges [1].

One noteworthy contribution to the field is the advent of silver nanoparticles (AgNPs), which have demonstrated remarkable antimicrobial efficacy in dentistry [2]. Integrated into various facets of dental care, silver nanoparticles exhibit potent antimicrobial, anti-inflammatory, and anti-oxidative properties. Their applications span from disinfection and prophylaxis to preventive measures against infections in the oral cavity. The synthesis of silver nanoparticles employs diverse methods, including chemical reduction and green synthesis utilizing plant-derived phenolic compounds [3].

In parallel, traditional medicinal plants such as *Ocimum basilicum *L.*, Ocimum gratissimum *L., and *Ocimum tenuiflorum *L. have garnered scientific attention for their diverse biological activities. Comparative extraction studies have highlighted the efficacy of acetone in yielding higher concentrations of phenolics, flavonoids, and tannins from these botanical sources [4]. Phytochemical analyses have unveiled a rich repertoire of bioactive compounds, including phenolic acids, flavonoids, and terpenes, affirming the medicinal potential of these plants [5]. The extracts derived from these Ocimum species have exhibited commendable antioxidant, antidiabetic, and anti-inflammatory activities. Notably, each species displays unique concentration-dependent attributes, with *Ocimum gratissimum *showcasing superior anti-inflammatory effects and *Ocimum tenuiflorum* exhibiting heightened antioxidant properties [6,7]. These findings not only validate the traditional use of these plants but also underscore their potential as therapeutic agents with multifaceted biological activities.

This study explores the synthesis of silver nanoparticles using herbal formulations derived from *Ocimum gratissimum* and *Ocimum tenuiflorum*, known for their diverse biological activities. The antimicrobial potential of the synthesized silver nanoparticles was assessed through the agar well diffusion technique and a time-kill curve assay.

## Materials and methods

Plant extract preparation

**Figure 1:**
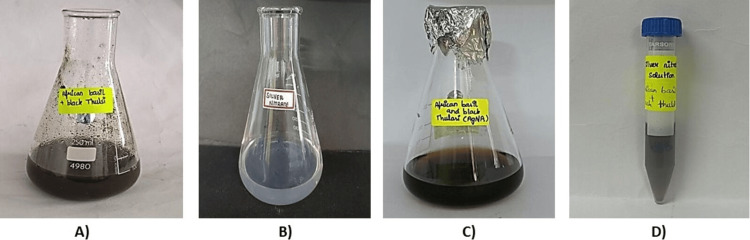
Green synthesis of silver nanoparticles using Ocimum tenuiflorum and Ocimum gratissimum herbal formulations. (A) Plant extract preparation; (B) Precursor solution (1 mm of silver nitrate in 70 mL distilled water); (C) Green-synthesized silver nanoparticle solution; (D) Silver nanoparticles collected after centrifugation process.

*Ocimum gratissimum* and *Ocimum tenuiflorum *extracts were prepared as follows: 2.5 g of African basil powder and 2.5 g of Black tulsi powder were mixed with 100 ml of double-distilled water and heated to a temperature range of 40 to 60 degrees Celsius for 15 to 20 minutes to create the aqueous extract [8]. This initial mixture resulted in a 1% plant extract solution. The extract was subsequently filtered and subjected to further heating until it was condensed to a final volume of 5 mL (Figure 1A).

Nanoparticle synthesis

Silver nanoparticles were prepared using the following method: A silver nitrate solution was obtained by dissolving 1 mm of silver nitrate in 70 mL of distilled water (Figure 1B). This silver nitrate solution (70 mL) was then mixed with the previously prepared *Ocimum gratissimum* and *Ocimum tenuiflorum* extract (30 mL) to initiate the synthesis of silver nanoparticles (Figure 1C). The solution was agitated at 250 rpm in an incubator until a discernible color change indicated the successful formation of nanoparticles [9]. Afterward, the solution was subjected to centrifugation at 8000 rpm for 10 minutes, and the resulting pellet was collected. To confirm the synthesis of silver nanoparticles at different time points (1, 12, 18, 24, 48, and 72 hours), the absorbance of the solution was measured using a UV-VIS spectrophotometer (ESICO). Subsequently, the solution was centrifuged at 10,000 rpm for 30 minutes (Figure 1D), and the pellet was dried in a hot air oven at 80°C for two hours. It was then rinsed with double-distilled water and 100% ethanol and stored in airtight containers for further analysis.

Antimicrobial activity

The antimicrobial activity of varying doses of silver nanoparticles was assessed against selected oral microbiomes, including *S. mutans, Lactobacillus acidophilus, C. albicans, Enterococcus faecalis,* and* S. aureus,* using the agar well diffusion method. Secondary cultures of microbial suspensions were evenly spread on both Mueller Hinton agar and Rose Bengal agar plates using a sterile spreader. Different concentrations of nanoparticles (25, 50, and 100 µL) were introduced into wells created on the agar plates using a sterile cork borer and a sterile micropipette. The concentration of silver nanoparticles in the solution was 10 mg per 100 ml. The plates were then incubated at 37°C for 24 to 48 hours, and the zone of inhibition (ZOI) in millimeters was measured and compared with the silver nanoparticle values. Each test was conducted in triplicate for analysis.

Time-kill curve assay

A time-kill curve assay was employed to evaluate the bactericidal qualities of *Ocimum gratissimum and Ocimum tenuiflorum*-infused silver nanoparticles and their concentration-dependent effects on the net growth rate of *S. mutans, Enterococcus faecalis, Staphylococcus aureus, Candida albicans, and Lactobacillus acidophilus*, at regular time intervals. The five pathogenic microorganisms were cultured in Mueller Hinton Broth, and various quantities of silver nanoparticles (25 µg, 50 µg, and 100 µg), plant extracts from *Ocimum gratissimum and Ocimum tenuiflorum* and controls (ampicillin for antibacterial and cycloheximide for antifungal) were added. The growth curves were generated after a four-hour preincubation period to ensure that all pathogens reached a stable early to mid-log phase. A 0.5 McFarland standard of each pathogen was inoculated into sterile phosphate-buffered saline [10]. This inoculum was derived from cultures that had been incubated at 37 degrees Celsius for 18 to 20 hours on Mueller Hinton agar plates. Subsequently, 30 mL of the inoculum was diluted in 15 mL of pre-heated antimicrobial-free Mueller Hinton Broth medium, and 90 mL of the resulting mixture was evenly distributed over each well of a 96-well ELISA plate. Ten microliters of silver nanoparticles at three different concentrations, along with plant extracts and untreated control, were added to each well containing 90 microliters of pre-incubated wound pathogens. A time-kill curve assay was then carried out to assess the antimicrobial properties [11,12].

Statistical analysis

Data were analyzed using IBM Corp., released in 2015 (IBM SPSS Statistics for Windows, Version 23.0., IBM Corp., Armonk, NY). The normality of the data was tested using the Kolmogorov-Smirnov and Shapiro-Wilk tests, which indicated a non-parametric distribution. To compare the absorbance between different treatment groups at various time intervals of 530 nm, the data were analyzed using measures such as mean, standard deviation, and percentages.

## Results

Antimicrobial activity

**Figure 2:**
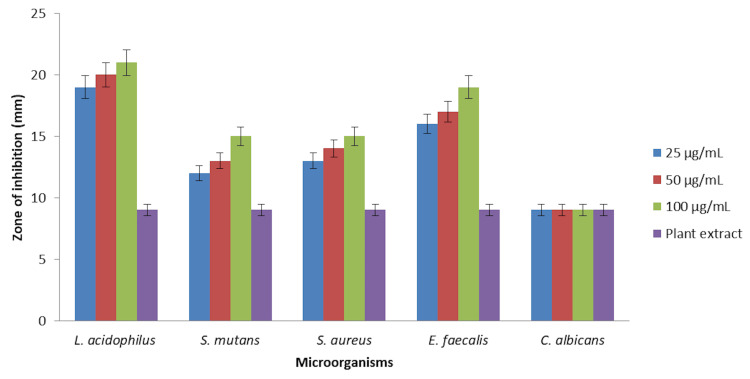
Antimicrobial activity of silver nanoparticles using agar well diffusion method. The error bars represent the standard error of mean.

In Figure 2, the green-synthesized silver nanoparticles demonstrated significant antibacterial activity at all concentrations tested. Against *S. mutans*, *S. aureus*, *E. faecalis*, and *Lactobacillus* sp., even the lowest dose of silver nanoparticles (25 µL) outperformed the control plant extract in terms of antibacterial activity. This suggests that the silver nanoparticles derived from *Ocimum tenuiflorum* and *Ocimum gratissimum* herbal formulations exhibit superior antimicrobial effects compared to the plant extract alone, emphasizing their efficacy against a spectrum of bacterial pathogens commonly associated with oral health.

In contrast, when assessing the activity against the fungal pathogen *C. albicans*, all concentrations of silver nanoparticles and the control plant extract demonstrated similar levels of antibacterial activity. This intriguing observation suggests a potential difference in the response of *C. albicans* to the silver nanoparticles compared to the bacterial strains tested. The results consistently revealed a concentration-dependent trend, with higher doses of silver nanoparticles exhibiting enhanced antibacterial activity against the bacterial strains. This is indicative of the dosage sensitivity of the silver nanoparticles in exerting their antimicrobial effects, underscoring the importance of dosage considerations in potential therapeutic applications [13]. Comparing the silver nanoparticles with the control plant extract highlights the enhanced antibacterial efficacy conferred by the nanoparticles. The superior performance of even the lowest silver nanoparticle dose suggests the potential of these nanoparticles as effective antimicrobial agents, surpassing the inherent antibacterial properties of the plant extract alone.

Time-kill curve assay

**Figure 3:**
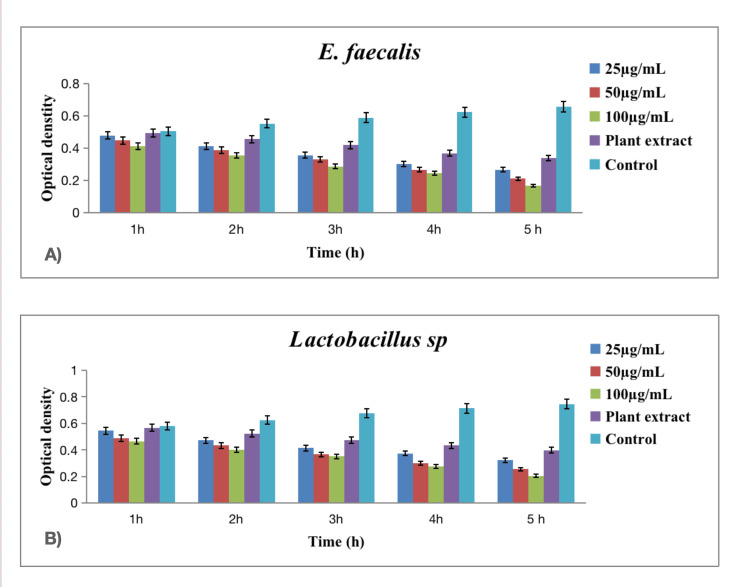
Time-kill assay graph of green-synthesized silver nanoparticles against various oral pathogens. (A) E. faecalis; (B) Lactobacillus sp. The error bar represents the standard error of mean.

**Figure 4:**
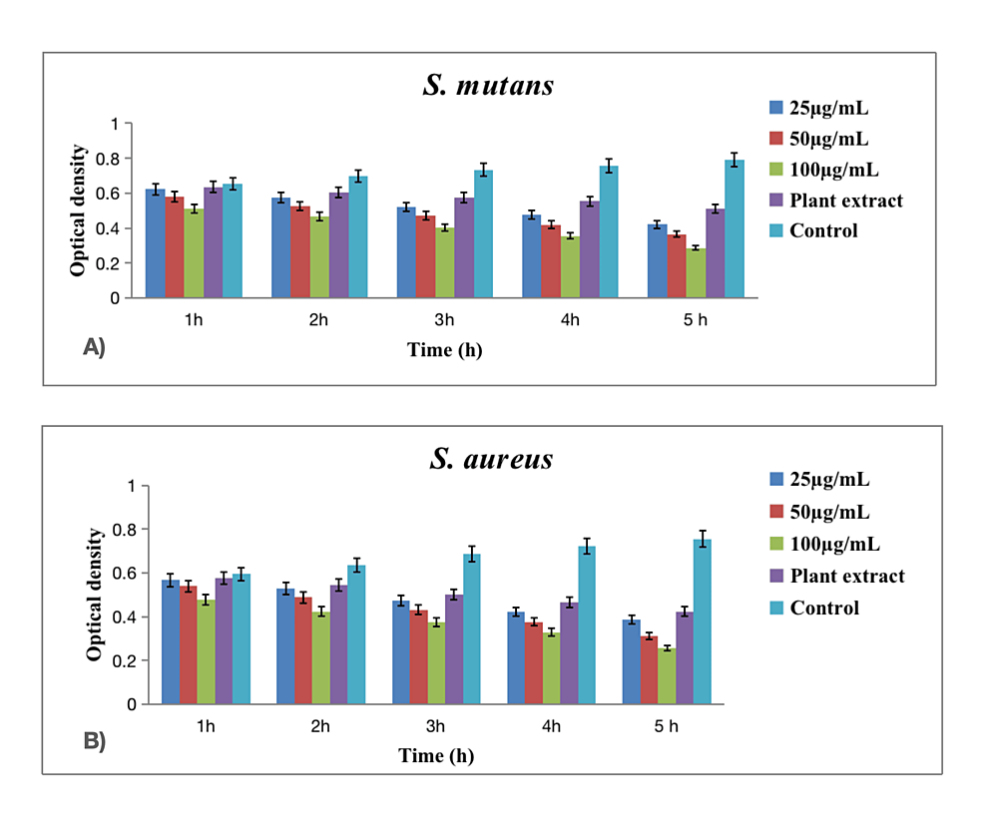
Time-kill assay graph of green-synthesized silver nanoparticles against various oral pathogens. (A) S. mutans and (B) S. aureus. The error bar represents the standard error of mean.

**Figure 5:**
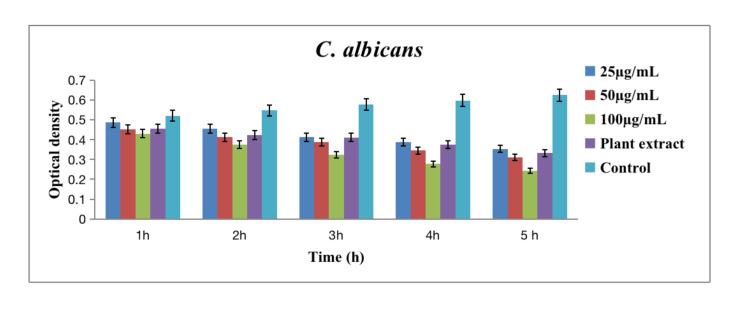
Time-kill assay graph of green-synthesized silver nanoparticles against C. albicans. The error bar represents the standard error of mean.

In Figures 3, 4, 5, time-kill curve assay was conducted to assess the antibacterial and antifungal activities of green-synthesized silver nanoparticles against various oral pathogens, including *Enterococcus faecalis* (*E. faecalis* (Figure 3A)), *Lactobacillus* sp. (Figure 3B), *Streptococcus mutans* (*S. mutans* (Figure 4A)), *Staphylococcus aureus* (*S. aureus *(Figure 4B)), and *Candida albicans* (*C. albicans* (Figure 5)). The assay spanned a five-hour period with measurements taken at hourly intervals and included three concentrations of silver nanoparticles (25 µg/mL, 50 µg/mL, and 100 µg/mL).

The results demonstrated a pathogen-specific response to the green-synthesized silver nanoparticles. Against *E. faecalis*, higher concentrations (50 µg/mL and 100 µg/mL) exhibited a significant reduction in bacterial growth over time, with the concentration of 100 µg/mL showing the most pronounced effect. *Lactobacillus* sp. displayed a similar concentration-dependent response, with the 100 µg/mL concentration resulting in the greatest reduction in bacterial growth. In the case of *S. mutans*, the trend was consistent, with higher concentrations leading to more substantial reductions in bacterial growth at all time points. The antibacterial activity against *S. aureus* followed a concentration-dependent pattern, with the highest concentration yielding the most significant reduction. Additionally, against *C. albicans*, the antifungal activity was concentration-dependent, with the highest concentration showing the most pronounced reduction in fungal growth [14].

Analyzing the data across hourly intervals and concentrations, at each hour, higher concentrations of silver nanoparticles consistently led to greater reductions in bacterial and fungal growth across all tested pathogens. The temporal effect was notable, with a progressive reduction in microbial growth observed over the five-hour assay period. Specifically, the concentration of 100 µg/mL demonstrated the highest efficacy in suppressing the growth of *E. faecalis*, *Lactobacillus* sp., *S. mutans*, *S. aureus*, and *C. albicans*. In this assay, the pathogens that consistently showed a high reduction in microbial growth across all concentrations and time points were *Staphylococcus aureus* and *Candida albicans*. Specifically, the concentration of 100 µg/mL demonstrated the most significant reduction in bacterial growth for *S. aureus* and fungal growth for *C. albicans* at each hour of the assay, indicating a robust antibacterial as well as antifungal effect against these pathogens. The plant extract alone exhibited some inherent antibacterial activity, and the control group values provided a baseline for microbial growth in the absence of silver nanoparticles.

## Discussion

The present study investigated the antimicrobial activities of green-synthesized silver nanoparticles derived from *Ocimum tenuiflorum* and *Ocimum gratissimum* herbal formulations against key oral pathogens, including *Streptococcus mutans* (*S. mutans*), *Staphylococcus aureus* (*S. aureus*), *Enterococcus faecalis* (*E. faecalis*), *Candida albicans* (*C. albicans*), and *Lactobacillus* sp. The results obtained through the agar well diffusion method and time-kill curve assay provide valuable insights into the effectiveness of these silver nanoparticles as antimicrobial agents [14].

The agar well diffusion method revealed significant antibacterial activity of green-synthesized silver nanoparticles at all concentrations tested against *S. mutans*, *S. aureus*, *E. faecalis*, and *Lactobacillus* sp. The superiority of even the lowest silver nanoparticles dose (25 µL) compared to the control plant extract suggests that the nanoparticles play a crucial role in enhancing antibacterial efficacy. The concentration-dependent trend observed further underscores the dosage sensitivity of silver nanoparticles in exerting their antimicrobial effects. This finding is consistent with previous studies indicating the enhanced antibacterial properties of silver nanoparticles [15,16]. Interestingly, against the fungal pathogen *C. albicans*, both silver nanoparticles and the control plant extract demonstrated similar levels of antibacterial activity. This observation suggests a potential difference in the response of *C. albicans* to silver nanoparticles compared to the bacterial strains tested. The consistent concentration-dependent trend across all bacterial strains indicates the broad-spectrum antibacterial potential of the silver nanoparticles, with higher doses consistently showing enhanced efficacy [17].

The time-kill curve assay further elucidated the pathogen-specific response to green-synthesized silver nanoparticles. Higher concentrations of silver nanoparticles (50 µg/mL and 100 µg/mL) exhibited a significant reduction in bacterial growth over time against *E. faecalis*, *Lactobacillus* sp., *S. mutans*, and *S. aureus*. The concentration of 100 µg/mL consistently demonstrated the most pronounced effect across these bacterial strains, indicating a concentration-dependent antibacterial activity [18]. The temporal effect observed in the assay, with a progressive reduction in microbial growth over the five-hour period, highlights the dynamic nature of silver nanoparticles in inhibiting bacterial proliferation. *Staphylococcus aureus* and *Candida albicans* consistently exhibited high sensitivity to silver nanoparticles, with the concentration of 100 µg/mL showing the most significant reduction in bacterial growth for *S. aureus* and fungal growth for *C. albicans* at each hour of the assay. Comparing the silver nanoparticles with the control plant extract emphasizes the enhanced antibacterial efficacy conferred by the nanoparticles.

Silver nanoparticles synthesized with *Ocimum tenuiflorum* demonstrate antimicrobial activity against various pathogens. The combination of silver nanoparticles reinforced with *O. tenuiflorum* and *Stevia rebaudiana* extracts exhibits efficacy against *Streptococcus mutans*, *Staphylococcus aureus*, *Lactobacillus* sp., and *Candida albicans* [19]. Another study involving silver nanoparticles synthesized with the leaf extract of *Catharanthus roseus* and *O. tenuiflorum* reveals inhibitory effects on *Escherichia coli* and *Bacillus subtilis*, with *O. tenuiflorum* silver nanoparticles displaying a more pronounced effect against *E. coli* [20]. Additionally, silver nanoparticles formed through the reduction of silver nitrate with aqueous mycelial extracts of *Ganoderma lucidum* exhibit antimicrobial effectiveness against both Gram-positive and Gram-negative bacteria, as well as yeasts [21]. Furthermore, the synthesis of AgNPs using mint and *O. tenuiflorum* demonstrates excellent antimicrobial activity, specifically against *Streptococcus mutans* [22]. In a previous study, it was identified that silver nanoparticles produced with *O. basilicum* plant extract effectively inhibited the growth of *Pseudomonas aeruginosa* and *Candida albicans* [23]. More et al. [24] also reported the antibacterial efficacy of silver nanoparticles synthesized using *O. basilicum* extract against *Escherichia coli*. Similarly, another research study revealed that AgNPs, synthesized with *O. basilicum* leaf extract, demonstrated antibacterial activity against *Escherichia coli*, *Enterococcus faecalis*, *Klebsiella pneumoniae*, and *Staphylococcus aureus *[25].

The findings suggest the potential of these silver nanoparticles as effective antimicrobial agents, surpassing the inherent antibacterial properties of the plant extract alone. The dosage sensitivity observed underscores the importance of precise dosing in potential therapeutic applications of silver nanoparticles [26]. The broad-spectrum antimicrobial activity against both bacteria and fungi, especially against *S. aureus* and *C. albicans*, positions these silver nanoparticles as promising candidates for oral health applications.

Limitations

A significant limitation of this study lies in its exclusive assessment of gram-positive bacteria and fungi for the antimicrobial activity of silver nanoparticles. This restricts the generalizability of findings and overlooks potential variations in susceptibility among gram-negative bacteria. To enhance the study's comprehensiveness, future research should expand its scope to include gram-negative bacteria, providing a more holistic understanding of silver nanoparticles antimicrobial efficacy. Additionally, the study could benefit from further exploration into the synthesis of nanoparticles using probiotic bacteria, offering valuable insights into their antibacterial activity against challenging pathogens and broadening the potential applications of silver nanoparticles in combating diverse microbial threats.

Implications for the population

The demonstrated antibacterial potential of green-synthesized silver nanoparticles, particularly against *Lactobacillus acidophilus* and *Enterococcus faecalis*, holds significant implications for the field of nanotechnology in medicine. As the demand for environmentally friendly and cost-effective resources continues to grow, the utilization of plant extracts in the green synthesis of nanoparticles becomes even more relevant and contributes to the global efforts to create biocompatible and economically viable nanoparticles with applications in medicine benefiting both the scientific community and the general population.

## Conclusions

In conclusion, the present study demonstrates the antimicrobial efficacy of a novel mixture of silver nanoparticles synthesized using *Ocimum tenuiflorum *and *Ocimum gratissimum* extract solution, and it may be developed as a promising antimicrobial agent against different oral pathogens.
